# Supplementary material for: Comparison of Nine Early Warning Scores for Identification of Short-Term Mortality in Acute Neurological Disease in Emergency Department
Source: J Pers Med. 2022 Apr 14;12(4):630. doi: 10.3390/jpm12040630 (PMC9024907; doi:10.3390/jpm12040630)
Supplement: Supplementary file 1 [file jpm-12-00630-s001.zip › jpm-1642584-supplementary.pdf]

Supplementary Table S1. AUROC, cut-off points for combined sensitivity and specificity with best score (Youden's test) for the different scales analyzed (for 7-, 14- and 21-day mortality).

Scores	Non-survivors 7-day	Non-survivors 14-day	Non-survivors 21-day
NEWS			
Cut-off	6	5	5
AUROC	0.863 (0.820-0.907)	0.849 (0.809-0.890)	0.820 (0.780-0.859)
Sensitivity	79.8 (71.5-86.2)	86.2 (79.7-90.9)	81.5 (75.1-86.6)
Specificity	77.3 (74.7-79.8)	68.9 (66.0-71.6)	69.6 (66.7-72.4)
PPV	27.7 (23.2-32.8)	28.3 (24.3-32.7)	32.0 (27.8-36.5)
NPV	97.2 (95.9-98.2)	97.2 (95.7-98.2)	95.5 (93.8-96.8)
Likelihood ratio +	3.52 (3.05-4.07)	2.77 (2.47-3.10)	2.68 (2.38-3.02)
Likelihood ratio -	0.26 (0.18-0.38)	0.20 (0.13-0.30)	0.27 (0.19-0.37)
Odds ratio	13.51 (8.36-21.82)	13.83 (8.47-22.58)	10.09 (6.72-15.16)
Diagnostic accuracy		71.0 (68.4-73.6)	71.4 (68.7-73.9)
ViEWS			
Cut-off	5	5	5
AUROC	0.862 (0.819-0.906)	0.849 (0.809-0.889)	0.818 (0.778-0.857)
Sensitivity	89.5 (82.5-93.9)	84.8 (78.1-89.8)	80.3 (73.8-85.6)
Specificity	69.9 (67.0-72.6)	71.0 (68.2-73.7)	71.8 (68.9-74.6)
PPV	24.5 (20.6-28.8)	29.5 (25.3-34.0)	33.3 (29.0-38.0)
NPV	98.4 (97.2-99.1)	97.0 (95.6-98.0)	95.4 (93.7-96.7)
Likelihood ratio +	2.97 (2.66-3.32)	2.93 (2.60-3.30)	2.85 (2.52-3.23)
Likelihood ratio -	0.15 (0.09-0.26)	0.21 (0.14-0.32)	0.27 (0.20-0.37)
Odds ratio	19.73 (10.69-36.40)	13.71 (8.54-22.02)	10.43 (6.99-15.55)
Diagnostic accuracy	71.8 (69.2-74.3)	72.8 (70.1-75.2)	73.1 (70.5-75.6)
MEWS			
Cut-off	4	3	4
AUROC	0.860 (0.816-0.904)	0.834 (0.793-0.876)	0.824 (0.784-0.863)
Sensitivity	77.2 (68.7-83.9)	85.5 (78.9-90.3)	69.4 (62.1-75.8)
Specificity	81.6 (79.2-83.9)	69.5 (66.6-72.2)	83.8 (81.4-86.0)
PPV	31.4 (26.3-37.1)	28.6 (24.5-33.0)	42.9 (37.2-48.7)
NPV	97.0 (95.7-98.0)	97.1 (95.6-98.1)	94.0 (92.2-95.4)
Likelihood ratio +	4.21 (3.58-4.95)	2.80 (2.50-3.14)	4.28 (3.60-5.09)
Likelihood ratio -	0.28 (0.20-0.39)	0.21 (0.14-0.31)	0.37 (0.29-0.46)
Odds ratio	15.05 (9.46-23.96)	13.43 (8.30-21.73)	11.70 (8.12-16.86)
Diagnostic accuracy	81.2 (78.9-83.4)	71.5 (68.8-74.0)	81.6 (79.3-83.8)
MREMS			
Cut-off	8	8	8
AUROC	0.889 (0.849-0.929)	0.882 (0.845-0.918)	0.866 (0.831-0.902)
Sensitivity	71.1 (62.1-78.6)	69.0 (61.0-75.9)	63.6 (56.2-70.4)
Specificity	89.3 (87.3-91.0)	90.8 (88.9-92.5)	91.6 (89.7-93.2)
PPV	42.0 (35.2-49.0)	51.8 (44.8-58.8)	57.0 (49.9-63.8)
NPV	96.6 (95.2-97.6)	95.3 (93.8-96.5)	93.5 (91.8-94.9)
Likelihood ratio +	6.64 (5.38-8.19)	7.53 (6.03-9.40)	7.56 (5.98-9.56)
Likelihood ratio -	0.32 (0.24-0.43)	0.34 (0.27-0.44)	0.40 (0.33-0.49)
Odds ratio	20.47 (13.06-32.09)	22.03 (14.60-33.24)	19.02 (12.97-27.88)
Diagnostic accuracy	87.5 (85.5-89.3)	88.1 (86.1-89.8)	87.4 (85.4-89.2)
EWS			
Cut-off	3	3	3
AUROC	0.851 (0.806-0.896)	0.836 (0.794-0.877)	0.818 (0.779-0.858)
Sensitivity	80.7 (72.5-86.9)	76.6 (69.0-82.7)	73.4 (66.4-79.4)
Specificity	78.2 (75.6-80.6)	89.7 (87.5-91.5)	80.4 (77.9-82.8)
PPV	28.8 (24.1-33.9)	54.4 (47.6-61.1)	39.7 (34.5-45.1)
NPV	97.4 (96.1-98.3)	96.0 (94.4-97.1)	94.5 (92.8-95.9)
Likelihood ratio +	3.70 (3.20-4.28)	7.40 (5.98-9.15)	3.75 (3.21-4.38)
Likelihood ratio -	0.25 (0.17-0.36)	0.26 (0.19-0.35)	0.33 (0.26-0.43)
Odds ratio	15.00 (9.21-24.44)	28.29 (18.22-43.94)	11.36 (7.83-16.48)
Diagnostic accuracy	78.4 (76.0-80.7)	87.8 (85.7-89.7)	79.4 (77.0-81.6)

HEWS			
Cut-off	4	4	4
AUROC	0.798 (0.748-0.848)	0.790 (0.745-0.836)	0.770 (0.727-0.813)
Sensitivity	77.2 (68.7-83.9)	75.9 (68.3-82.1)	71.7 (64.5-77.9)
Specificity	68.6 (65.8-71.4)	77.2 (74.4-79.8)	70.4 (67.5-73.2)
PPV	21.2 (17.5-25.3)	34.5 (29.5-39.9)	29.8 (25.6-34.4)
NPV	96.5 (94.9-97.6)	95.3 (93.5-96.6)	93.4 (91.4-95.0)
Likelihood ratio +	2.46 (2.15-2.82)	3.33 (2.87-3.87)	2.42 (2.12-2.77)
Likelihood ratio -	0.33 (0.24-0.47)	0.31 (0.23-0.42)	0.40 (0.31-0.51)
Odds ratio	7.41 (4.69-11.70)	10.66 (7.07-26.07)	6.02 (4.21-8.61)
Diagnostic accuracy	69.5 (66.8-72.1)	77.0 (74.4-79.5)	70.6 (67.9-73.2)
SEWS			
Cut-off	3	3	3
AUROC	0.851 (0.806-0.896)	0.836 (0.794-0.877)	0.818 (0.779-0.858)
Sensitivity	80.7 (72.5-86.9)	76.6 (69.0-82.7)	73.4 (66.4-79.4)
Specificity	78.2 (75.6-80.6)	79.4 (76.8-81.8)	80.4 (77.9-82.8)
PPV	28.8 (24.1-33.9)	34.7 (29.7-40.1)	39.7 (34.5-45.1)
NPV	97.4 (96.1-98.3)	96.0 (94.4-97.1)	94.5 (92.8-95.9)
Likelihood ratio +	3.70 (3.20-4.28)	3.72 (3.20-4.32)	3.75 (3.21-4.38)
Likelihood ratio -	0.25 (0.17-0.36)	0.30 (0.22-0.40)	0.33 (0.26-0.43)
Odds ratio	15.00 (9.21-24.44)	12.59 (8.33-19.03)	11.36 (7.83-16.48)
Diagnostic accuracy	78.4 (76.0-80.7)	79.1 (76.7-81.3)	79.4 (77.0-81.6)
RAPS			
Cut-off	3	4	3
AUROC	0.832 (0.785-0.879)	0.821 (0.778-0.864)	0.810 (0.770-0.851)
Sensitivity	82.5 (74.4-88.3)	70.3 (62.5-77.2)	78.0 (71.3-83.6)
Specificity	69.4 (66.5-72.1)	80.3 (77.7-82.6)	71.7 (68.8-74.5)
PPV	22.7 (18.9-27.0)	33.8 (28.7-39.3)	32.6 (28.3-37.3)
NPV	97.3 (95.3-98.3)	95.0 (93.3-96.3)	94.9 (93.1-96.3)
Likelihood ratio +	2.70 (2.38-3.05)	3.57 (3.03-4.20)	2.76 (2.43-3.13)
Likelihood ratio -	0.25 (0.17-0.38)	0.37 (0.29-0.48)	0.31 (0.23-0.41)
Odds ratio	10.66 (6.47-17.58)	9.67 (6.55-14.26)	9.02 (6.13-13.26)
Diagnostic accuracy	70.7 (68.0-73.2)	79.1 (76.6-81.3)	72.7 (70.0-75.2)
WPSS			
Cut-off	3	3	3
AUROC	0.825 (0.777-0.873)	0.817 (0.774-0.860)	0.790 (0.748-0.831)
Sensitivity	95.6 (90.1-98.1)	93.8 (88.6-96.7)	90.2 (84.8-93.8)
Specificity	55.8 (52.8-58.8)	57.1 (54.1-60.2)	58.0 (54.8-61.0)
PPV	19.1 (16.1-22.5)	23.8 (20.5-27.5)	27.3 (23.8-31.1)
NPV	99.2 (98.0-99.6)	98.5 (97.1-99.2)	97.1 (95.4-98.2)
Likelihood ratio +	2.16 (2.00-2.34)	2.19 (2.02-2.38)	2.14 (1.96-2.34)
Likelihood ratio -	0.08 (0.03-0.19)	0.11 (0.06-0.21)	0.17 (0.11-0.27)
Odds ratio	27.56 (11.15-68.10)	20.15 (10.15-40.01)	12.65 (7.55-21.20)
Diagnostic accuracy	59.7 (56.9-62.5)	61.7 (58.9-64.5)	62.8 (59.9-65.5)

Bracketed numbers indicate 95% confidence interval. NEWS: National Early Warning Score; ViEWS: Vital PAC Early Warning Score; MEWS: Modified Early Warning Score; MREMS: Modified Rapid Emergency Medicine Score; EWS: Early Warning Score; HEWS: Hamilton Early Warning Score; SEWS: Standardised Early Warning Score; RAPS: Rapid Acute Physiology Score; WPSS: WHO Prognostic Scored System; AUROC: area under the receiver operating characteristics; PPV: positive predictive value; NPV: negative predictive value.

Supplementary Table S2. Comparison of the AUROC of the different scores (ICU admissions)

Scores	NEWS	ViEWS	MEWS	MREMS	EWS	HEWS	SEWS	RAPS	WPSS
NEWS	1	0.713	0.059	0.463	0.634	**0.005**	0.636	0.251	**0.000**
ViEWS	0.713	1	**0.046**	0.500	0.562	**0.007**	0.564	0.233	**0.000**
MEWS	0.059	**0.046**	1	**0.033**	**0.033**	**0.000**	**0.033**	0.964	**0.000**
MREMS	0.463	0.500	**0.033**	1	0.241	0.131	0.241	**0.001**	**0.046**
EWS	0.634	0.562	**0.033**	0.241	1	**0.000**	0.387	0.286	**0.000**
HEWS	**0.005**	**0.007**	**0.000**	0.131	**0.000**	1	**0.000**	**0.000**	0.431
SEWS	0.636	0.564	**0.033**	0.241	0.387	**0.000**	1	0.285	**0.000**
RAPS	0.251	0.233	0.964	**0.001**	0.286	**0.000**	0.285	1	**0.000**
WPSS	**0.000**	**0.000**	**0.000**	**0.046**	**0.000**	0.431	**0.000**	**0.000**	1

NEWS: National Early Warning Score; ViEWS: Vital PAC Early Warning Score; MEWS: Modified Early Warning Score; MREMS: Modified Rapid Emergency Medicine Score; EWS: Early Warning Score; HEWS: Hamilton Early Warning Score; SEWS: Standardised Early Warning Score; RAPS: Rapid Acute Physiology Score; WPSS: WHO Prognostic Scored System.
Values in bold differ from 0 with a significance level of alpha = 0.05.

Supplementary Table S3. Comparison of the AUROC of the different scores (2-day mortality)

Scores	NEWS	ViEWS	MEWS	MREMS	EWS	HEWS	SEWS	RAPS	WPSS
NEWS	1	0.777	0.619	0.215	0.350	**0.011**	0.349	0.781	**0.000**
ViEWS	0.777	1	0.574	0.201	0.414	**0.013**	0.414	0.828	**0.000**
MEWS	0.619	0.574	1	0.273	**0.019**	**0.001**	**0.019**	0.499	**0.000**
MREMS	0.215	0.201	0.273	1	**0.030**	**0.002**	**0.030**	**0.025**	**0.000**
EWS	0.350	0.414	**0.019**	**0.030**	1	**0.023**	0.522	0.702	**0.006**
HEWS	**0.011**	**0.013**	**0.001**	**0.002**	**0.023**	1	**0.023**	0.085	0.321
SEWS	0.349	0.414	**0.019**	**0.030**	0.522	**0.023**	1	0.701	**0.006**
RAPS	0.781	0.828	0.499	**0.025**	0.702	0.085	0.701	1	**0.032**
WPSS	**0.000**	**0.000**	**0.000**	**0.000**	**0.006**	0.321	**0.006**	**0.032**	1

NEWS: National Early Warning Score; ViEWS: Vital PAC Early Warning Score; MEWS: Modified Early Warning Score; MREMS: Modified Rapid Emergency Medicine Score; EWS: Early Warning Score; HEWS: Hamilton Early Warning Score; SEWS: Standardised Early Warning Score; RAPS: Rapid Acute Physiology Score; WPSS: WHO Prognostic Scored System.
Values in bold differ from 0 with a significance level of alpha = 0.05.
